# Corrigendum: Examining Associations Between Psychopathic Traits and Executive Functions in Incarcerated Violent Offenders

**DOI:** 10.3389/fpsyt.2020.575349

**Published:** 2020-09-10

**Authors:** Carl Delfin, Peter Andiné, Björn Hofvander, Eva Billstedt, Märta Wallinius

**Affiliations:** ^1^Department of Psychiatry and Neurochemistry, Centre for Ethics, Law and Mental Health, Institute of Neuroscience and Physiology, Sahlgrenska Academy, University of Gothenburg, Gothenburg, Sweden; ^2^Research and Development Unit, Regional Forensic Psychiatric Clinic, Växjö, Sweden; ^3^Forensic Psychiatric Clinic, Sahlgrenska University Hospital, Gothenburg, Sweden; ^4^Department of Forensic Psychiatry, National Board of Forensic Medicine, Gothenburg, Sweden; ^5^Department of Clinical Sciences Lund, Child, and Adolescent Psychiatry, Faculty of Medicine, Lund University, Lund, Sweden; ^6^Gillberg Neuropsychiatry Centre, Institute of Neuroscience and Physiology, University of Gothenburg, Gothenburg, Sweden

**Keywords:** psychopathy, executive functions, neuropsychological tests, offenders, violence, crime, prison

In the original article, there was an error. We recently discovered that one offender had participated twice in the study, with the second participation while serving a new sentence for a violent crime within the same region but in the end of the inclusion period for the study. This was discovered when we performed registry-based follow-ups using the offenders’ social security numbers. The second participation of this offender was removed from the data, and thus, the number of participants in our study is decreased from 214 to 213. All data have been re-analyzed using the new sample, with only minor differences. None of these differences affect the scientific conclusions in any way. In addition, there was a typing error in the published article that incorrectly stated that the male offenders in the sample were aged 18-25, when it should correctly state that they were 19-25.

The corrected paragraphs are below, with changes highlighted in bold:

The **Abstract**:

“**Two hundred and thirteen** incarcerated male violent offenders were assessed with the Psychopathy Checklist-Revised and completed tests of cognitive flexibility, spatial working memory, response inhibition, and planning and problem-solving using the Cambridge Neuropsychological Test Automated Battery.”

The **Materials and Methods** section, subsection **Participants**:

“Participants (**N = 213**) were male violent offenders recruited from the Development of Aggressive Antisocial Behavior Study (DAABS). The DAABS recruited young adult male offenders (aged 18–25 years at inclusion) who were convicted of hands-on violent (including sexual) offenses and imprisoned in one out of nine prisons in the western region of the Swedish Prison and Probation Service between March 2010 and July 2012, with a participation rate of 71%. All assessments were based on file reviews, structured clinical interviews, self-report, observations, and neuropsychological testing. Interviews, observations, and neuropsychological testing were administered during a full day by a clinical psychologist with special training in the methods used. Detailed descriptions of the cohort are provided in previous publications (50–52). In the total DAABS cohort (**N = 269**), 54 offenders did not participate in or complete all the neuropsychological assessments used in this study, and psychopathy ratings were unavailable for two offenders. Thus, the current study sample consisted of **213** male offenders, aged **19–25** at the time of inclusion (**M = 21.93**, SD = 1.87).”

The **Materials and Methods** section, subsection **Measures**, paragraph 1 “Psychopathic Traits”:

Psychopathic traits were measured using the PCL-R (39), which consists of 20 items rated on a three-point scale (0 = does not apply, 1 = may apply or applies in some respects, 2 = does apply). We adopted the four-facet structure of the PCL-R, in which possible scores for facets 1 (interpersonal traits) and 2 (affective traits) ranges from 0 to 8, and possible scores for facets 3 (lifestyle traits) and 4 (antisocial traits) ranges from 0 to 10. The offenders were rated by an experienced and for the task specifically trained psychologist based on all information available from interviews, observations, and files. Training sessions with consensus ratings on participants, led by an experienced PCL-R assessor, were performed to ensure inter-rater reliability. The mean PCL-R total score in the study sample was **17.51 (SD = 7.06**). Internal consistency was good, with Cronbach’s alpha (α) = 0.85 for the total score being slightly above pooled estimates from the PCL-R Technical Manual (53). Cronbach’s α was 0.65, 0.81, 0.78, and 0.77 for the interpersonal, affective and lifestyle facets, respectively, indicating lower but adequate internal consistency. The mean corrected item-total correlation for the total score was 0.47, with 0.56, 0.70, 0.64, and 0.61 interpersonal, affective, lifestyle, and antisocial facets, respectively, also in line with the PCL-R Technical Manual. Note that item N ranged from 204 to **213** for the total score, and 210 to **213** for the facets.

The corrected **Table 1** appears in this article, with changes marked in bold.

**Table 1:** Descriptive statistics (*N* = 213).

	Mean ± SD	Range
PCL-R interpersonal facet score	0.9 ± 1.34	0-8
PCL-R affective facet score	**3.16** ± 2.26	0-8
PCL-R lifestyle facet score	**6.43** ± 2.61	0-10
PCL-R antisocial facet score	**6.28** ± 2.88	0-10
IED stages completed	**8.11** ± 1.13	1-9
IED errors	**26.65 ± 12.51**	7-63
SWM errors	23.14 ± **17.28**	0-90
SWM strategy score	**32.5 ± 5.11**	0-47
SST stop-signal RT	0.19 ± 0.08	0.07-0.74
SST mean correct RT	**0.49** ± 0.14	0.3-1.27
SOC MITT	6.03 ± **4.79**	0-29.38
SOC problems solved	8.3 ± 1.75	4-12

SD, standard deviation; PCL-R, Psychopathy Checklist–Revised; IED, Intra/Extra Dimensional Shift; SWM, Spatial Working Memory; SST, Stop-Signal Task; RT, reaction time; SOC, Stockings of Cambridge; MITT, mean initial thinking time.

The corrected **Figure 1** appears in this article. Changes are minor, with most occurring on second decimal level (e.g., posterior probability of the correlation between SOC MITT and PCL-R lifestyle facet changes from 0.9 to 0.89).

**Figure 1:**
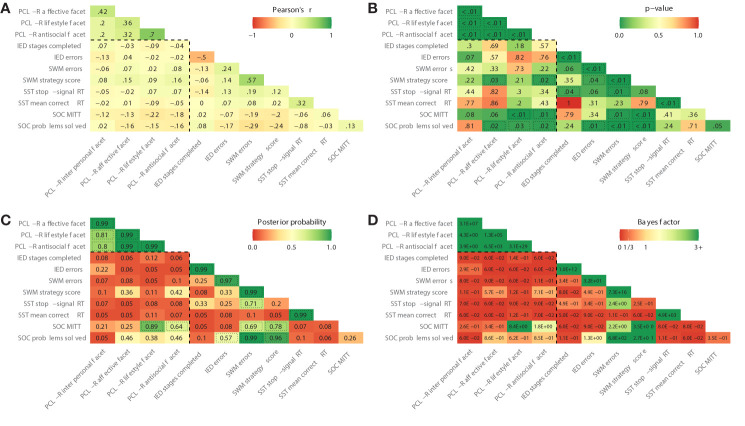
**(A–D)** Zero-order correlations (Pearson’s r), p-values, posterior probabilities and Bayes factors. The dashed box contains the primary study variables. Dotted lines indicate statistical significance at p < 0.05 **(B)** or a posterior probability > 0.50 **(C)**. PCL-R, Psychopathy Checklist-Revised; IED, Intra/Extra- Dimensional Shift; SWM, Spatial Working Memory; SST, Stop-Signal Task; RT, reaction time; SOC, Stockings of Cambridge; MITT, mean initial thinking time.

The authors apologize for this error and state that this does not change the scientific conclusions of the article in any way.

## Conflict of Interest

The authors declare that the research was conducted in the absence of any commercial or financial relationships that could be construed as a potential conflict of interest.

The handling editor declared a past collaboration with the authors.

